# Has the association between low school performance and the risk of disability benefit due to mental disorders become stronger over time?

**DOI:** 10.1186/s12889-019-6703-7

**Published:** 2019-04-03

**Authors:** Beata Jablonska, Christina Dalman, Andreas Lundin, Kyriaki Kosidou

**Affiliations:** 1 Centre for Epidemiology and Community Medicine, Stockholm County Council, Box 45436, 104 31 Stockholm, Sweden; 2 Department of Public Health Sciences, Karolinska Institutet, 171 77 Stockholm, Sweden

**Keywords:** Disability benefits, Mental disorders, School performance, Cohort studies, Epidemiology, Sweden

## Abstract

**Background:**

The proportion of young adults on disability benefits due to mental disorders has increased in Europe since the early 2000’s. Poor educational achievement is a risk factor for disability benefits due to mental disorders in early adulthood, yet no study has examined whether this association has become stronger over time.

**Methods:**

All residents of Stockholm County at the time of graduation from compulsory education between 2000 and 2007 (*N* = 169,125) were followed prospectively for recipient of disability benefits due to a mental disorder from 2003 to 2011. Information about the study participants was obtained by linkage of national registers. Low school performance in the last year of compulsory school was defined as having a merit rating corresponding to the lowest quintile. The association between school performance and disability benefits was examined using Cox proportional hazards models.

**Results:**

Low school performers had a greater risk of disability benefits due to mental disorders during early adulthood, as compared to their better performing counterparts, and this association was more pronounced for the more recent graduation cohorts (OR = 1.12, 95% CI 1.08–1.16).

**Conclusions:**

The association between low school performance and the risk of disability benefits due to mental disorders seems to become stronger during the first decade of the twenty-first century. It is plausible that this trend indicates an increased vulnerability of poor school performers to exclusion from the labor market. Prevention of school failure and adjustment of the labour market to individual variability in academic performance appear to be critical approaches to counteract this trend.

**Electronic supplementary material:**

The online version of this article (10.1186/s12889-019-6703-7) contains supplementary material, which is available to authorized users.

## Background

The prevalence of disability benefits among young adults has increased sharply in many European countries since the early 2000’s [1, 2]. From 2005 through 2015, the incidence of activity compensation, i.e. a specific disability benefit paid to young adults ages 19–29 who are not able to work full time for at least one year due to longstanding illness, injury or disability, increased in Sweden from 3,5 to approximately 6,2 per 1000 [1]. Mental disorders are the most common diagnosis group among young disability beneficiaries [2, 3]. In many cases young people enter the disability benefit system without ever entering the workforce and stay on benefits for their lifetime [1].The cause of the increases in disability benefits among young adults is not known but could be related to the observation that disparities in health and labor market opportunities between education groups have recently increased [4–7].

Employment opportunities increasingly lie in jobs requiring at least secondary level qualifications mainly because the number of low skilled jobs is shrinking and expected to continue doing so in Sweden and in other OECD countries [8, 9]. Furthermore, the number of highly educated that is forced to perform low-skilled jobs has also grown in Sweden, thus further reducing the employment opportunities of the low-educated [10]. During the study period the unemployment rate of low-educated youth increased in Sweden from 20,9% in 2003 to 38,4% in 2011 [11]. The large oversupply of low-skilled workers in relation to demand (11 job seekers per job vacancy compared to 1.6 per job vacancy where upper secondary education is required), means that many low educated individuals are increasingly disadvantaged since they match poorly with the needs of the Swedish labor market.

In Sweden and other western countries, poor school performance in compulsory school is an early indicator of later poor mental health [12] and a determinant of future educational outcomes and employment status in early adulthood [13]. Previous research has found that poor educational outcomes, including low school performance in compulsory school, are positively associated with the risk of disability benefits due to mental disorders in early adulthood [14–16]. Further, characteristics of the labor market such as rapid technological development and increased competition for low skilled occupations may have deterred employment prospects among young people with low academic qualifications, with increasing mental health problems and disability benefits among this group as the consequence. However, no study has yet examined whether the association between poor educational outcomes and the risk for disability pension in early adulthood has become stronger over time.

The aim of the current study was to investigate whether the association between low school performance and the risk of disability benefits due to mental disorders has become stronger during the early twenty-first century. We hypothesized that young adults with low school performance at the time of leaving compulsory school would have a greater risk of disability benefits due to mental disorders during early adulthood, as compared to their better performing counterparts, and that this association would be more pronounced for the more recent graduation cohorts.

## Methods

The present prospective cohort study is based on data from the Psychiatry Sweden register linkage, a unique database which contains information from a range of Swedish health and administrative registries. Data from these registries were linked by using the 10-digit personal identification number assigned to all individuals with permanent residence in Sweden.

The study was conducted in Stockholm County, the largest county in Sweden in terms of population (approximately 2.2 million). This study was approved by the regional ethical review board in Stockholm, Sweden (Dnr 2010/1185-31/5). The present study is based on retrospective analysis of registry data. For this type of study formal consent is not required.

### Study population

All residents of Stockholm County at the time of graduation from compulsory education (i.e., 9th grade) between 2000 and 2007 (*N* = 169,125) were followed prospectively in the National Social Insurance Agency’s database MicroData for Analyses of the Social insurance (MiDAS) for recipient of disability benefits due to a mental disorder from 2003 to 2011. Subjects were censored at the date of death (retrieved from the National Cause of Death Register), date of emigration (retrieved from the Register of the Total Population) or at the end of follow-up. Persons with diagnoses of organic mental disorders (F06-F09) or mental retardation (F70-F79) (*n* = 153) were excluded. Furthermore, 4773 persons were not available for the follow-up due to emigration or death before 19 years of age, leaving the final sample of 164,293 individuals.

### Outcome

The outcome was defined as first-time disability benefits with a diagnosis of mental disorder during follow-up 2003–2011, and obtained from MiDAS. Psychiatric diagnoses were recorded according to the 10th revision of the International Classification of Diseases (ICD-10) [17] and grouped into five diagnostic categories: schizophrenia and non-affective psychoses (F20–29), mood disorders (F30–39), neurotic and somatoform disorders (F40–F48), pervasive and specific developmental disorders (F80-F89), and behavioral and emotional disorders with onset usually occurring in childhood and adolescence (F90–F98).

In Sweden, adults between 19 and 29 years of age can receive disability benefits if their work capacity is chronically reduced with at least 25% due to medical reasons. Individuals with a disease that have incomplete schooling can also be permitted disability benefits as an allowance for continuing schooling, in order to finish elementary or secondary school. We were not able to distinct between these two reasons for receiving disability benefits due to disease in our data.

### Explanatory variables

#### Graduation cohort

Graduation cohort was defined as a one-year graduation group, based on date of graduation from compulsory school, ranging from 2000 to 2007, collected from The National School Register.

#### School performance

Data on school performance were collected from The National School Register. Low school performance in the last year of compulsory school, when the children were about 16 years old, was defined as having a merit rating (MR) corresponding to the lowest quintile of MR, calculated on the basis of the minimum of eight completed courses, or having nine or more incomplete courses.

The MR for a student who left compulsory school with finished courses was during the study period the sum of the grades for his/her 16 best grades, where G (pass) = 10 scores, VG (pass with distinction) = 15 scores, and MVG (pass with special distinction) = 20 scores, yielding a maximum possible score of 320. The MR for subjects that a student has not completed successfully is equal to zero.

#### Confounders

Mental disorders before finishing ninth grade and country of birth were examined as possible confounders, since both factors have been associated with school performance [18] and mental health in adulthood [19]. Mental disorders before finishing ninth grade were defined as any registered ICD-10 F00–99 diagnosis in the Stockholm Child and Adolescent Psychiatric Care Register (child and adolescent psychiatric inpatient and outpatient care within Stockholm County since 2001) [20], The VAL database (public health care services provided by Stockholm County since 1997), and The National Patient Register (discharge diagnoses of all inpatient since 1973 and specialist outpatient care since 2001 in Sweden) [21].

Country of birth was derived from Statistics Sweden’s Register of the Total Population and grouped into *Sweden* or *other country*.

### Statistical methods

The association between school performance and disability benefits was examined using Cox proportional hazards regression modelling. Follow-up time was calculated as the number of days between turning 19**,** which was the youngest age for registration of disability benefits, and date of receiving disability benefits, migrating, dying or until end of follow-up on December 31, 2011. Associations are reported as hazard ratios (HR) with 95% confidence intervals.

The analysis for the full sample was carried out in four modes: 1) model with graduation cohort as explanatory variable, 2) model with low school performance as explanatory variable stratified by graduation cohort, 3) model with graduation cohort as explanatory variable stratified by school performance, 4) model with an interaction term between graduation cohort and school performance. All models were initially adjusted for country of birth, but this had very limited impact on the estimates and was thus not included in the final models. Five additional models were fitted where the effect of the interaction between graduation cohort and merit rating was tested for each mental disorders diagnostic group separately. All these models were also estimated separately by gender**.**

The proportional hazards assumption, i.e. that the survival curves for two strata of school performance must have hazard functions that are proportional over time, was evaluated using log-log survival functions separately for each graduation cohort. There was no evidence for non-proportionality of considered log-log survival curves. Since disability benefits can be granted to individuals with a disease who have not completed their schooling, sensitivity analyses were performed where individuals who had nine or more incomplete courses, and therefore, presumably, the target group of receiving disability benefits for schooling, were excluded. Due to limited number of cases, sensitivity analyses were not performed for specific mental disorders diagnostic group.

## Results

In total, 2237 individuals received disability benefits due to a mental disorder during the follow up period 2003–2011. In Table 1, HR and CI for disability benefits in relation to graduation cohort are shown. The risk of disability benefits increased gradually for younger graduation cohorts compared to the reference cohort that graduated on year 2000.

**Table 1:** Compulsory school graduation cohort and risk of disability benefits due to mental disorders during follow-up 2003–2011

Graduation cohort	All		Men		Women	
	HR^a^	95%CI	HR^a^	95%CI	HR^a^	95%CI
2000 (*n* = 17,542)	1.00		1.00		1.00	
2001 (*n* = 18,289)	1.03	0.87–1.23	1.05	0.83–1.33	1.01	0.79–1.30
2002 (*n* = 19,114)	1.18	0.99–1.40	1.08	0.85–1.38	1.29	1.01–1.64
2003 (*n* = 19,518)	1.24	1.04–1.48	1.32	1.03–1.67	1.16	0.90–1.51
2004 (*n* = 21,063)	1.54	1.30–1.83	1.65	1.30–2.08	1.43	1.11–1.84
2005 (*n* = 21,881)	1.56	1.30–1.90	1.74	1.37–2.21	1.34	1.03–1.75
2006 (*n* = 23,472)	1.74	1.45–2.08	1.73	1.35–2.22	1.75	1.35–2.28
2007 (*n* = 23,414)	2.15	1.79–2.58	1.99	1.55–2.57	2.32	1.78–3.03

^a^hazard ratio

Low school performance was strongly associated with disability benefits and the strength of the association appeared to grow for each younger graduation cohort. The increases were less pronounced in models that took account of mental disorder before finishing ninth grade (for details, see the Additional file 1).

Table 2 shows the association between graduation cohort and disability benefits due to mental disorders within low and higher performers. Younger graduation cohorts had an elevated risk of disability benefits, compared to the 2000 graduation cohort, regardless of school performance. However, the risk increase was more pronounced among individuals with poor school performance. Adjustment for mental disorders before graduation abolished the cohort effect within the higher performing group. However, the effect remained significantly higher in the low performance group, albeit diluted.

**Table 2:** Compulsory school graduation cohort and risk of disability benefits due to mental disorders during follow-up 2003–2011, stratified by school performance at the time of leaving compulsory school

	Low school performance^a^		Higher school performance^b^		Low school performance^a,c^		Higher school performance^b,c^		Low school performance^a,d^		Low school performance^a,c,d^	
	HR^e^	95%CI	HR^e^	95%CI	HR^e^	95%CI	HR^e^	95%CI	HR^e^	95%CI	HR^e^	95%CI
2000/01	1.00		1.00		1.00		1.00		1.00		1.00	
2002/03	1.26	1.07–1.50	1.14	0.96–1.37	0.97	0.82–1.15	0.93	0.78–1.11	1.21	1.01–1.46	0.93	0.77–1.12
2004/05	1.78	1.50–2.11	1.38	1.14–1.66	1.19	1.01–1.41	0.97	0.80–1.17	1.77	1.47–2.13	1.19	1.00–1.43
2006/07	2.35	1.97–2.79	1.55	1.26–1.91	1.38	1.15–1.65	0.95	0.77–1.18	2.49	2.06–3.00	1.45	1.20–1.76

^a^defined as the lowest quintile of merit rating (calculated on the basis of at least 8 school subjects) or ≥ 9 incomplete courses at the time of leaving compulsory school

^b^defined as merit rating above the lowest quintile

^c^adjusted for mental disorders before finishing ninth grade

^d^individuals who had ≥9 incomplete courses excluded (*n* = 8589)

^e^hazard ratio

There was a significant interaction between graduation cohort and school performance regarding risk of disability benefits (Table 3). The strength of this association was only marginally attenuated by adjustment for mental disorders before graduation. In sensitivity analyses, exclusion of individuals having nine or more incomplete courses produced the same pattern of results.

**Table 3:** Interaction effects between graduation cohort and school performance at the time of leaving compulsory school on risk of disability benefits due to mental disorders during follow-up 2003–2011

	Model 1		Model 1a		Model 2		Model 2a	
	HR^b^	95%CI	HR^b^	95%CI	HR^b^	95%CI	HR^b^	95%CI
Graduation cohort * school performance (higher school performance^a^ as a reference)	1.11	1.07–1.15	1.09	1.05–1.13	1.12	1.08–1.16	1.10	1.06–1.14

^a^ merit rating above the lowest quintile

^b^ hazard ratio

Model 1poor school performance defined as the lowest quintile of merit rating (calculated on the basis of at least 8 school subjects) or ≥ 9 incomplete courses at the time of leaving compulsory school

Model 1a adjusted for mental disorders before finishing ninth grade

Model 2 individuals who had ≥9 incomplete courses excluded (*n* = 8589)

Model 2a adjusted for mental disorders before finishing ninth grade

*Interaction between two variables

Additional analyses of specific psychiatric diagnostic groups revealed that the interaction between graduation cohort and school performance regarding risk of disability benefits was particularly pronounced for psychotic disorders and mood disorders among women, and for pervasive and specific developmental disorders among men (Table 4).

**Table 4:** Interaction effects between graduation cohort and school performance at the time of leaving compulsory school on risk of disability benefits due to specific mental disorders during follow-up 2003–2011

	ICD-10 code	All^b^		Men^b^		Women^b^	
		HR^c^	95%CI	HR^c^	95%CI	HR^c^	95%CI
Graduation cohort * low school performance (higher school performance^a^ as a reference)							
Schizophrenia, schizotypal, delusional, and other non-mood psychotic disorders (*n* = 197)	F20-F29	1.09	0.95–1.25	0.92	0.77–1.11	1.39	1.10–1.74
Mood disorders (*n* = 254)	F30–39	1.16	1.04–1.30	1.10	0.92–1.31	1.21	1.04–1.41
Anxiety, dissociative, stress-related, somatoform and other nonpsychotic mental disorders (*n* = 304)	F40–F48	1.10	0.99–1.23	1.15	0.97–1.36	1.06	0.93–1.22
Pervasive and specific developmental disorders (*n* = 837)	F80-F89	1.07	1.01–1.14	1.09	1.01–1.18	1.07	0.96–1.19
Behavioural and emotional disorders with onset usually occurring in childhood and adolescence (*n* = 402)	F90–F98	0.99	1.01–1.14	0.99	0.86–1.14	0.98	0.84–1.14

^a^merit rating above the lowest quintile

^b^poor school performance defined as the lowest quintile of merit rating (calculated on the basis of at least 8 school subjects) or ≥ 9 incomplete courses at the time of leaving compulsory school

^c^hazard ratio

*Interaction between two variables

## Discussion

Prior research suggests that poor school performance is a risk factor for recipient of disability benefits due to mental disorders later in life [14–16]. By following children in Stockholm County who graduated from the compulsory school between 2000 and 2007, our study both confirms and extends previous findings by demonstrating that the impact of low school performance on risk of disability benefits due to mental disorders appears to have increased across younger graduation cohorts. Our findings appear to be compatible with the observation that disparities in health between education groups have recently increased [4–6] but at odds with the conclusion of the Swedish study where the increasing importance of intelligence as a predictor of early disability pension among men between 1971 and 2006 has not been confirmed [22]. Although intelligence is a significant prerequisite for educational success [23], educational achievement is a multidimensional measure, that reflects both cognitive and non-cognitive abilities [24] and, as suggested before [25, 26], its predictive validity in terms of academic and labor market success, health and longevity may be better than that of tests measuring cognitive skills.

Our results also indicate that, selection mechanisms may contribute to the association between school performance and disability benefits since the association was attenuated by controlling for mental disorders before finishing ninth grade in our study. Development of mental health problems early in life might limit a child’s educational achievements and, either independently or in synergy with poor educational achievements, increase the risk of disability benefits later in life. According to The Schools Inspectorate, the agency responsible for supervision and quality assurance in regard to compulsory schools, the lack of individual support for students with special educational needs or disabilities is an issue for many schools [27]. Yet, control for mental disorders before finishing ninth grade did not attenuate the interaction between graduation cohort and school performance in our study and thus cannot explain our finding of an increased strength of the association between low school performance and disability benefits during the study period.

In all probability there has been no or little increase, in the proportion of ninth-graders who: did not meet the knowledge requirements laid down for one or more school subjects, were eligible for upper secondary school according to eligibility requirements 1998–2010, and in the transition rate to upper secondary school during the study period [28], suggesting a relatively constant level of this specific risk factor for disability benefits in early life. However, the possibility that the consequences of poor academic skills upon individuals’ vulnerability for mental disorders have changed over time as a result of environmental factors, such as labor market, educational system or medical praxis, cannot be ruled out.

It has previously been suggested that the influence of educational merits on chances of employment has become increasingly important during later years [7]. Limited employment prospects at the time of graduation have been found to increase mental and physical ill health later in life [29]. The association could be especially true for low school performers since they are disproportionally affected by poor labor market conditions. According to Eurostat youth unemployment rates in Sweden increased from 8 to 15% among those with upper secondary/post-secondary (non-tertiary) education during the first decade of the twenty-first century, whereas corresponding numbers among those with less than primary/primary/lower secondary education were higher, 15 and 37%, respectively [11]. Although not restricted to poor performers, the regional statistics showing the positive association between unemployment and disability benefits rates among youth seem to support this explanation [30].

Furthermore, low school performers are more likely to be employed on less secure temporary contracts [31], which have been found to increase the risk of mental health problems and disability benefits [32, 33]. In Sweden, as in many countries the proportion of temporary employees has increased in the labor market, especially among young people [30].

It could by hypothesized that changes in the education system, such as rising individualization of teaching in Sweden and elsewhere may, too, have contributed to our results. The current, student-driven, curriculum emphasizes students’ responsibility for directing, organizing and evaluating their own learning [34]. However, the transfer of responsibility for the student’s educational outcomes from teachers to students may lead to self-blame among students who failed to meet school expectations. Maladaptive emotion regulation strategies such as self-blame, may put a person at increased risk for mental health problems [35].

An alternative explanation for the likely greater role of low school performance on risk of disability benefits due to mental disorders could be an increased societal tendency to describe and treat a person’s shortcomings in functioning within the modern labor market and societal arena in general as medical ones [36, 37]. At a time of increasing pressure to be perfect, e.g. happy, good-looking, smart, have many friends [34, 38], and of less stigmatizing attitude toward seeking mental health services [39, 40], medical help may be seen as a remedy when failing to meet the internal or external expectations put on young people by society. Professionals, on the other hand, may be more prone to label personal and social issues as medical disorders that render one occupationally disabled, especially if the patient’s skill profile does not match the current labor market requirements [41]. Future studies may seek to address these explanations with empirical evidence.

### Strengths and limitations

This study has several strengths. First, the study covered the total Stockholm County population that graduated from compulsory school during the period from 2000 to 2007 and that was eligible for follow up in national registers during the period from 2003 to 2011. Second, the study was based on register data that are highly complete, which minimizes selection and attrition bias.

Yet, some limitations need to be kept in mind when interpreting the results. We were not able to distinguish between disability benefits permitted due to reduced working capacity and for prolonged schooling. It is probable that low school performers are overrepresented among those receiving compensation for prolonged schooling. However, it is important to underline that many of those granted disability benefits for prolonged schooling apply for and receive compensation for long-term reduced working capacity directly after heaving ended a period with compensation for schooling [42]. We have accounted for this, at least partly, by re-running analyses without individuals who had more than eight incomplete courses and who presumably, are eligible for prolonged schooling. The pattern of results is in both cases the same.

Although we controlled for mental disorders before finishing ninth grade the register-based design of this study did not allow for the control of mental health conditions that do not lead to formal help seeking. Thus, the reverse association, meaning that poor school performance may be a consequence of mental health problems cannot be entirely excluded.

Although the proportion of youth performing poorly at school has remained fairly constant during the study period, in absolute numbers the size of subsequent graduation cohorts, and thus the number of poor performers, has grown. Thus, being part of a larger graduation cohort may, in itself, be a disadvantage since members of large cohorts face greater competition for economic and social prosperities [43]. This, again, may doubly affect low performers – both by the general effect of competition and by the specific effect of these circumstances on low performers. We don’t know whether such a trend could have influenced our results. Average merit rating is one of several possible measures of school performance and since the Swedish grading system is highly decentralized, some effects of subjectivity in the grading process cannot be ruled out.

Finally, countries may differ in the level of support provided to the individuals with low school performance to enter the labor market which may, in turn, influence their risk of mental health problems and disability. The characteristics of the labor market such as level of competition, unemployment rates and availability of low skill occupations may also impact on the association between school performance and the risk of disability benefits. Thus, it is not certain whether the study results can be generalized to national contexts outside the Scandinavian welfare states.

## Conclusions

The influence of low school performance on risk of disability benefits due to mental disorders appears to have become stronger during the first decade of the twenty-first century. If this trend is related to the characteristics of the modern labor market, which we find likely, the exclusion of young people with poor school merits from the labor market may be even more pronounced in the future as we are entering an era when advanced technologies will additionally reduce the job supply and polarize the workforce.

Disability benefits are one of the principal achievements of the welfare state. Nevertheless, an unnecessary early exit from the labor market should always be prevented due to its deleterious effects on health and prosperity. To combat the increasing risk of disability benefit due to mental disorders among young adults with poor school results, there is an urgent need for policy makers to explore how working life and social policy should be modified or expanded to reduce the loss of this substantial human capital. In fact, for the majority of people with mental health problems employment has been shown to be beneficial [44]. Thus, the focus of existing policies on what the potential recipient of disability benefits cannot do should be balanced against the assessment of his/her capacity.

Prevention of school failure, at the first place, and the opportunity for *a second chance* for *the failures*, e.g. by allowing them to upgrade their qualifications so as to enhance their self-esteem, employability and competitiveness in the labor market, appear to be critical approaches to counteract this negative trend. Such low-threshold services based on the collaboration between municipalities, the non-profit sector, social and labor market authorities and that promote employability of excluded young people are already offered in Sweden but could be enriched and developed.

## Additional file

Additional file 1: **Table S1.** Low school performance at the time of leaving compulsory school and risk of disability benefits due to mental disorders during follow-up 2003–2011 in different graduation cohorts. **Table S2.** Interaction effects between graduation cohort and school performance as a continuous variable at the time of leaving compulsory school on risk of disability benefits due to mental disorders during follow-up 2003–2011. (DOCX 15 kb)
