# Supplementary material for: Has the association between low school performance and the risk of disability benefit due to mental disorders become stronger over time?
Source: BMC Public Health. 2019 Apr 3;19:376. doi: 10.1186/s12889-019-6703-7 (PMC6448315; doi:10.1186/s12889-019-6703-7)
Supplement: Supplementary file 1 — Table S1. Low school performance at the time of leaving compulsory school and risk of disability benefits due to mental disorders during follow-up 2003–2011 in different graduation cohorts. Table S2. Interaction effects between graduation cohort and school performance as a continuous variable at the time of leaving compulsory school on risk of disability benefits due to mental disorders during follow-up 2003–2011. (DOCX 15 kb) [file 12889_2019_6703_MOESM1_ESM.docx]

Supplementary tables

Supplementary Table 1 shows the association between school performance at the time of leaving compulsory school and the risk of disability benefits due to mental disorders within each graduation cohort. Low school performance was strongly associated with disability benefits and the strength of the association appeared to grow for each younger graduation cohort. The increases were less pronounced in models that took account of mental disorder before finishing ninth grade (Models 1a and 2a).

Supplementary Table 1. Low school performance at the time of leaving compulsory school and risk of

disability benefits due to mental disorders during follow-up 2003-2011 in different graduation cohorts.

| Graduation cohort | Model 1  HR^1^ (95%CI) | Model 1a  HR^1^ (95%CI) | Model 2  HR^1^ (95%CI) | Model 2a  HR^1^ (95%CI) |
| --- | --- | --- | --- | --- |
| 2000 | 4.44 (3.48-5.66) | 3.11 (2.79-4.62) | 4.10 (3.18-5.29) | 3.40 (2.61-4.42) |
| 2001 | 3.49 (2.74-4.44) | 2.66 (2.07-3.42) | 3.40 (2.54-4.36) | 2.63 (2.03-3.40) |
| 2002 | 4.08 (3.23-5.15) | 2.68 (2.10-3.42) | 3.83 (3.00-4.88) | 2.54 (1.97-3.26) |
| 2003 | 4.46 (3.51-5.66) | 2.63 (2.05-3.37) | 3.95 (3.07-5.08) | 2.35 (1.81-3.05) |
| 2004 | 5.26 (4.21-6.57) | 3.06 (2.43-3.86) | 4.87 (3.84-6.14) | 2.93 (2.28-3.70) |
| 2005 | 5.77 (4.55-7.32) | 3.52 (2.76-4.50) | 5.37 (4.19-6.88) | 3.33 (2.59-4.30) |
| 2006 | 7.07 (5.55-9.02) | 3.61 (2.81-4.62) | 6.78 (5.29-8.71) | 3.51 (2.72-4.53) |
| 2007 | 7.55 (5.87-9.71) | 4.05 (3.11-5.19) | 7.57 (5.86-9.79) | 4.10 (3.16-5.33) |

^1^hazard ratio

Model 1 poor school performance defined as the lowest quintile of merit rating (calculated on the basis of at least 8 school subjects) or ≥9 incomplete courses at the time of leaving compulsory school (reference: merit rating above the lowest quintile)

Model 1a adjusted for mental disorders before finishing ninth grade

Model 2 individuals who had ≥9 incomplete courses excluded (n=8589)

Model 2a adjusted for mental disorders before finishing ninth grade

Supplementary Table 2 shows the interaction effects between graduation cohort and school performance as a continuous variable. There was a significant interaction between graduation cohort and school performance regarding risk of disability benefits; the better the school performance the lower the risk of disability benefits. The strength of this association was slightly attenuated by adjustment for mental disorders before graduation

Supplementary Table 2. Interaction effects between graduation cohort and school performance as a continuous variable at the time of leaving compulsory school on risk of disability benefits due to mental disorders during follow-up 2003-2011.

|  | Model 1 | Model 1a |
| --- | --- | --- |
|  | HR^1^ 95%CI | HR^1^ 95%CI |
| Graduation cohort * school performance | 0.97 0.96-0.98 | 0.98 0.97-0.99 |

^1^ hazard ratio

Model 1a adjusted for mental disorders before finishing ninth grade
